# Parameters influencing health-related quality of life after severe trauma: a systematic review (part II)

**DOI:** 10.1007/s00068-023-02276-y

**Published:** 2023-05-15

**Authors:** Annesimone Lotfalla, Jens Anthony Halm, Tim Schepers, Georgios Fredericus Giannakópoulos

**Affiliations:** Department of Trauma Surgery, Trauma Unit, Amsterdam University Medical Center, Location Academic Medical Center, Meibergdreef 9, 1105 AZ Amsterdam, The Netherlands

**Keywords:** Health-related quality of life, Injuries, Literature review, Severe trauma, Patient-outcome assessment, Patient-reported outcomes, Polytrauma, Predictors, QoL assessment

## Abstract

**Introduction:**

It is increasingly recognized that health-related quality of life (HRQoL) is a relevant outcome to study in populations comprising severely injured patients. Although some studies have readily demonstrated a compromised HRQoL in those patients, evidence regarding factors that predict HRQoL is scarce. This hinders attempts to prepare patient-specific plans that may aid in revalidation and improved life satisfaction. In this review, we present identified predictors of HRQoL in patients that have suffered severe trauma.

**Methods:**

The search strategy included a database search until the 1st of January 2022 in the Cochrane Library, EMBASE, PubMed, and Web of Science, and reference checking. Studies were eligible for inclusion when (HR)QoL was studied in patients with major, multiple, or severe injury and/or polytrauma, as defined by authors by means of an Injury Severity Score (ISS) cut-off value. The results will be discussed in a narrative manner.

**Results:**

A total of 1583 articles were reviewed. Of those, 90 were included and used for analysis. In total, 23 possible predictors were identified. The following parameters predicted reduced HRQoL in severely injured patients and came forward in at least more than three studies: higher age, female gender, lower extremity injuries, higher rate of injury severity, lower achieved educational level, presence of (pre-existing) comorbidities and mental illness, longer duration of hospital stay, and high level of disability.

**Conclusion:**

Age, gender, injured body region, and severity of injury were found to be good predictors of health-related quality of life in severely injured patients. A patient-centered approach, based on individual, demographic, and disease-specific predictors, is highly recommended.

**Supplementary Information:**

The online version contains supplementary material available at 10.1007/s00068-023-02276-y.

## Introduction

Traumatic injuries continue to represent one of the leading causes of mortality and morbidity worldwide [1, 2]. Although the survival rate of trauma patients has increased over the last decades for multiple reasons [3–11], the consequences of the acquired injuries still influence multiple important life domains, including the physical, psychological, and social domain [12], indicating the limiting ability of survival and death to describe the impact of traumatic injuries accurately. These concepts are captured in the definition of health-related quality of life (HRQoL), which is defined by the United States Food and Drug Administration (FDA) as following [13]:

“A multidomain concept that represents the patient’s general perception of the effect of illness and treatment on physical, psychological, and social aspects of life.”

From this definition, it becomes clear that HRQoL is a personal, qualitative, and certainly broad concept. Multiple HRQoL models have therefore been developed to guide research, the so-called ‘conceptual models’ [14]. These models aid in understanding and providing the variables that are associated with HRQoL [15–18], such as individual, demographic, and environmental characteristics, but also disease-specific and biological parameters.

Although HRQoL remains difficult to investigate, despite these useful models, research into long-term outcomes (so-called patient-reported outcome measures, or PROMs) has become of increasing interest during the last decade due to improvements in trauma patient mortality rates. This has shifted the research focus from mortality to morbidity, disability, and the influence on life satisfaction. A promising development, since information regarding a trauma survivors’ pathway to recovery in the domains that the definition of HRQoL encompasses, is still scarce. The latter is especially the case in severely injured patients, a group of patients for whom HRQoL is a subject that demands more attention in research since quantification of severe trauma impact on patients in the long-term and evidence regarding post-injury state of these patients is yet incompletely unraveled.

Populations that require comprehensive care due to the potentially life-threatening and/or disabling injuries sustained, are patients suffering severe injury, major trauma, or poly-trauma. In literature, these terms are often used interchangeably, and, internationally, various definitions by means of the Injury Severity Score (ISS) seem to be well accepted for these populations [19]. In those patients, a higher rate of mortality is demonstrated [20–22]. Now that severely injured patients are surviving their injuries more often as well, and hence are more at risk at living with the sequels of their acquired injuries, surgeries, and treatments, it is to be expected that this group will express reduced HRQoL. This has been affirmed in a previous review [23], in which most studies found diminished scores assessed by several HRQoL-instruments when compared to normative population values and pre-injury status. Although this is valuable information, optimization and advancements are hard to accomplish without information regarding factors that predict and determine HRQoL [24].

The prediction of one’s individual HRQoL after severe traumatic consequences is relevant and may aid in preparation of patient-specific plans [25]. To our knowledge, no systematic review has been performed to date that presents those predictors in a comprehensive manner for the population of severely injured patients in specific. This hinders attempts to prevent reduction of HRQoL, or at least to improve HRQoL after severe trauma. Earlier reviews [23, 26, 27] have focused on instruments used for HRQoL-assessment in trauma patients and HRQoL outcomes after injury but have not included predictors.

Considering the abovementioned gaps in reports and paucity of systematically reviewed data regarding factors impacting HRQoL among severely injured patients, establishment of predictors of HRQoL is indispensable for hospital and rehabilitation management and personalized, patient-centered care. In this review, we aim to identify the predictors of HRQoL in severely injured patients.

## Methods

### Search strategy

An exhaustive search was conducted in the Cochrane Library, EMBASE, PubMed, and Web of Science covering the population of severely injured patients and outcome of interest (i.e., HRQoL). All articles published from inception to the 1st of January 2022 were imported in Rayyan QCRI [28] . In addition to database-searched articles, references of (Cochrane) reviews and included articles were checked and screened for title and abstract by two reviewers.

The applied search strategies were adjusted to the syntax appropriate for that database and are reported as full texts in Supplementary Appendix A. There were no restrictions with regard to the year of publication.

### Eligibility criteria

Studies that met the following criteria were included:

I. Studies have the objective to describe predictors (and not merely associations) of QoL or HRQoL (since both terms are used interchangeably in literature) of patients who have suffered major trauma, severe injury, or poly-trauma according to authors’ own given definition that is well described by means of an ISS threshold value (e.g., defined as a population ISS ≥ 16—other thresholds used to define major or severe trauma, however, were accepted as well, since we aimed to cover literature’s HRQoL data on severe trauma patients, taking into account the different ISS thresholds that exist in literature for severely injured patients).
II. The publication is an original article.
III. The article is published in English, German, French, Arabic, or Dutch (language selection based on the authors’ language skills).
IV. The full text of the article is available.

Studies on general injury populations and injury-specific studies were both included. Multiple independent populations included in one study were considered separately.

The following articles were excluded:

I. Studies that included exclusively patients described as having suffered mild or minor trauma.
II. Studies using exclusively trauma severity indices scores other than ISS.
III. Studies not defining the patient-reported outcome (PRO) explicitly in terms of (HR)QoL, but in less standardized terms, such as disability, (dys)function, or (dis)satisfaction.
IV. Books, commentaries, letters, reports, (conference) abstracts, posters, presentations, discussion papers, (systematic) reviews, editorials.

Application of the eligibility criteria was done in duplicate.

### Data extraction

Data extraction from the included studies was done in duplicate to avoid errors and missing relevant data. From each eligible study, the following data-elements were extracted into a Microsoft Excel sheet:

- Study characteristics: first author’s last name, year of publication, geographical location, study design, number of participants.
- Population characteristics: age at baseline, gender, median/mean ISS, general population description (e.g., mechanism of injury), duration of ICU and hospital stay.
- PRO characteristics: reported PRO, instrument used to assess PRO, elements observed with instrument, follow-up duration/time point of outcome assessment.
- Outcome: (HR)QoL predictors, results summarization, author conclusion.

### Synthesis of results

A narrative design was chosen for this review, since a meta-analysis could not be produced due to the heterogeneity (e.g., in the used instruments and study population) of the included studies. Factors possibly associated with HRQoL will be discussed in a qualitative manner.

## Results

### Literature search

**Fig. 1:**
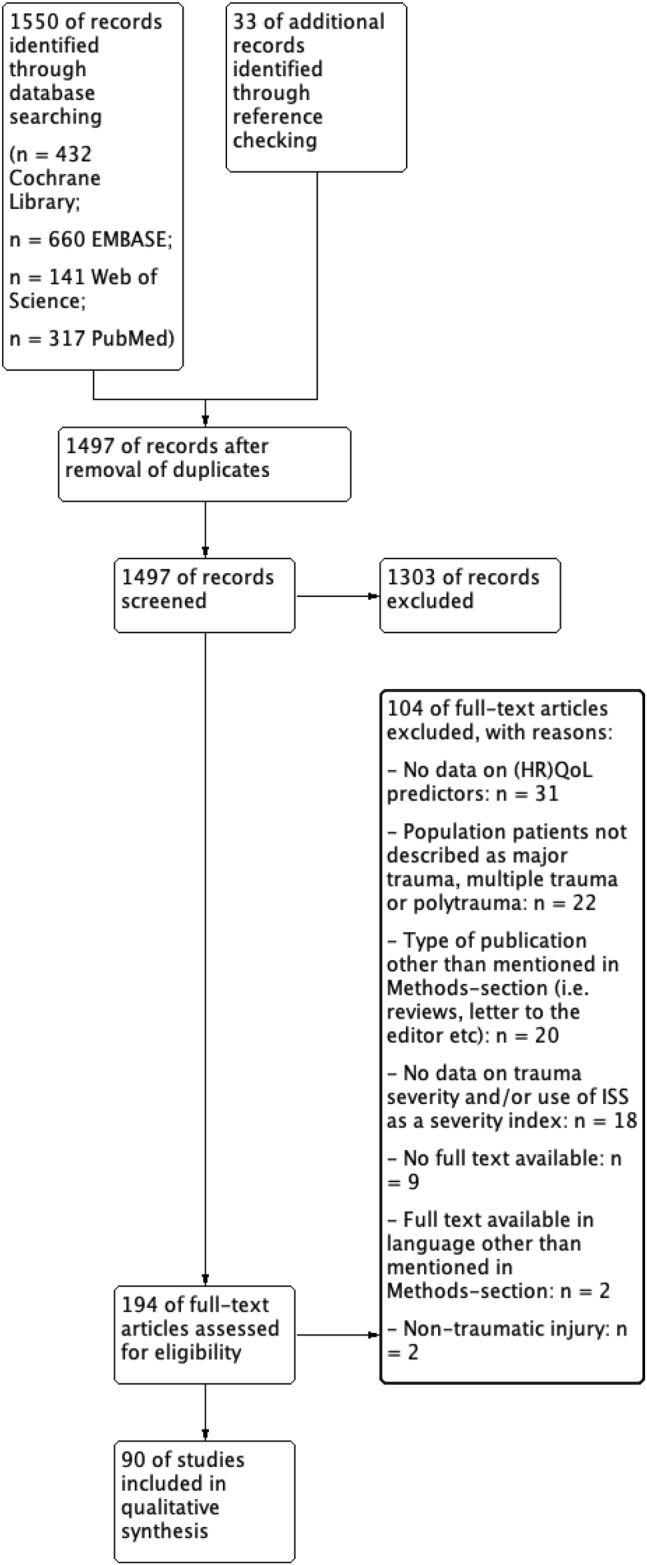
Flowchart of study inclusion during the search review process

The search strategy (literature search and reference checking) identified 1583 articles of potentially relevant articles, of which 194 remained after an initial screening of titles and abstracts and removal of duplicates. Application of the eligibility criteria on the full-text papers, resulted in inclusion of 90 articles for final analysis. The main reasons for exclusion of publications were the lack of data on HRQoL predictors and the inclusion of populations not defined as having suffered severe trauma. The study flow diagram is shown in Fig. 1**.**

### General study characteristics

In total, 90 studies were included, all published between January 1994 and May 2022. The general characteristics of the studies are presented in Table 1 (Supplementary Appendix B).

The majority of studies were conducted prospectively (*n* = 52) [2, 29–40, 50–52, 54, 56–59, 61–72, 77, 78, 80, 86, 130, 133, 136, 137, 139, 142, 146–148, 150–154, 157] and in Germany (*n* = 23) [35, 39, 41–44, 51, 52, 57, 62–65, 71, 73–75, 79, 80, 83, 86, 152, 154]. The average ISS in studies ranged from 9 to 57.

Sample sizes ranged between 22 [60] and 1238 [149], with the majority of patients being male (> 50% in 83 articles [2, 29–44, 46–48, 50–56, 58, 59, 61–75, 77–86, 130, 131, 133–159]). Average age of the participants in the included studies ranged between 7 [159] and 68 [153] years. Four studies [45, 133, 136, 159] assessed HRQoL in children and/or adolescents (≤ 18 years) exclusively.

All of the included studies have identified predictors or variables that could be associated with the HRQoL of severely injured patients. These will be discussed in the following section and are summarized in Table 1 (Supplementary Appendix B). The parameters could be subdivided into the following categories: patient-specific (sociodemographic and disease-related), injury-specific, hospital-related, and other parameters (i.e., the factors that do not fit into one of the other categories).

#### Patient-specific parameters: sociodemographic

I. **Age**: In 38 studies [2, 29–49, 133, 134, 136, 138, 140, 144–151, 153, 154, 157], the relationship between age and HRQoL was reported. The majority of those studies (*n* = 24) found a significant correlation with age, mainly indicating that older age was an independent predictor of reduced HRQoL [2, 29, 31, 33, 35–37, 41, 42, 44, 133, 134, 136, 138, 145–147, 149, 153, 154, 157], disablement [46, 152], and use of pain medication [149]. In some studies, the cut-off age for poor HRQoL was further specified: worse HRQoL was demonstrated in patients ≥ 55 [31, 138, 150], ≥ 60 [29], > 65 [139, 148, 154] and > 80 years [146, 147].
  Pediatric patients presented with comparable HRQoL to healthy peers and didn’t demonstrate any significant HRQoL reduction [29, 45, 136]. Teenagers and adolescents were at risk for poorer HRQoL in comparison to younger children [133, 136].
  Patients with severe traumatic brain injury (TBI) or spinal cord injuries who were younger at the time of injury were more likely to demonstrate poor HRQoL [48, 136]. One study [37], however, reported a better self-reported physical health two years post-TBI in younger aged patients.
II. **Gender**: Gender was investigated as a determinant for HRQoL in 24 studies [32, 34–37, 40, 41, 43, 47–53, 58, 68, 133, 138, 139, 141, 150, 151, 158]. Twenty studies [34–37, 40, 41, 47, 48, 50–53, 68, 133, 138, 139, 141, 150, 151, 158] found a significant correlation between gender and HRQoL, the majority of which (*n* = 16) [34–36, 41, 48, 50–52, 68, 133, 138, 139, 141, 150, 151, 158] indicated that female gender was a predictor for worse HRQoL, even in adolescents [133].
  In their patient population who underwent open reduction and internal fixation (ORIF) of the pubic symphysis after poly-trauma, Giannoudis et al. [47] reported worse HRQoL in men in all age groups compared to women. Men were also less likely to return to work post-injury [32, 40].
III. **Education**: Patients who had achieved a lower educational level (according to the used definitions in the different countries) reported a reduced HRQoL [31, 35, 37, 41, 54, 68, 149] and more severe pain in the long term [54, 149]. Patients with higher education returned more often to work [32, 40].
IV. **Socioeconomic status (SES)**: A lower socio-economic status (SES) predicted reduced HRQoL according to several 36-Item Short Form Survey (SF-36) subscales, such as bodily pain and mental health [44].
V. **Social network**: A sufficient social network was demonstrated to have a significant impact on satisfaction with life and return to work [32, 82], as do households composed of more than one person [138]. Participation in society predicted physical health as measured by the Physical Component Score (PCS) as well as mental health and social functioning two years post-trauma [67, 68].
VI. **Work**: Reduced HRQoL was associated with reduced capacity to work [54] and vice versa did unemployment, inability to work, and having to deal with financial loss due to trauma predict a poorer HRQoL [32, 35, 41, 42, 86, 144, 158]. The capacity to return to work impacted HRQoL positively, and improved HRQoL helped patients return to work [155]. Employed patients were less likely to report poor HRQoL compared to unemployed patients at the time of injury [35, 37]. Unemployment was an independent predictor for use of pain medication in patients’ one-year post-trauma [149].
  A higher Mental Component Score (MCS) made it more likely for patients to return to their main occupation [49], while low self-reported general health predicted problems regarding return to work [152]. A longer stay in the hospital or rehabilitation institute made it less likely to return to work, as did a lower self-reported coping [40]. Higher educated patients and patients with a white-collar job (also known as suit-and-tie or office workers) were more likely to return to work [32, 40]. Patients not returning to work after discharge reported more pain and worse self-reported functioning [40]. Blue collar professions (i.e., those who do manual labor) were associated with long-term pain severity [54].

#### Patient-specific parameters: disease-related

I. **Body Mass Index (BMI):** A BMI ≥ 25 was a predictor of a lower EQ-VAS [31]. A greater BMI was associated with more severe pain in the long term [54] (see Table 1, Supplementary Appendix B).
II. **Comorbidity**: Significantly lower HRQoL scores were found in patients with pre-injury comorbidity (not further specified) [31, 36, 41, 55, 138–140, 155, 158]. Pain medication was significantly less used in patients without pre-existing comorbidities [149]. Pre-existing comorbidities did not predict patients returning to work [152].
III. **Psychiatric illness**: Patients presenting with symptoms of posttraumatic stress disorder (PTSD) after severe trauma reported poorer HRQoL [30, 33, 49]. PTSD severity predicted the height of HRQoL scores, both physical and mental [33, 49]; patients with full PTSD presented with the poorest HRQoL, followed by patients with partial PTSD [84].
  Patients presenting with psychologic distress or previous mental health issues were at risk of poor HRQoL [33, 63, 76, 86, 142, 145, 149], as well as depressed patients as assessed by various depression measuring scales [37, 42, 130]. Mental health predicted MCS even after 10 years [67]. Preinjury, current drug, or alcohol disorders were associated with poorer HRQoL and daily pain and use of pain medication on the long term [145, 149].
  Patients who did not (completely) return to work, presented with higher levels of depression, anxiety, PTSD, and ill mental functioning [144].
IV. **Cognitive function:** Patients with mental or cognitive impairment or dysfunction reported lower HRQoL scores in several subscales, such as financial loss and loss of friends, while individuals without impairments and a strong sense of coherence present with better HRQoL and life satisfaction [43, 68, 82]. Cognitive functioning was correlated with physical health [67, 68] and return to work [40].

#### Injury-specific parameters

I. **Body region of injury**: 47 publications [2, 29–31, 34–39, 42–44, 46, 48, 53–55, 60, 63–76, 86, 130–133, 136, 140–143, 149, 155, 156, 159] reported on the influence of the location of injury on the HRQoL.
  Injury to the extremities was an important predictor for poor HRQoL [29–31, 35, 39, 42, 44, 70, 72–76, 131, 132, 142, 143, 149, 155, 156], with multiple extremity injuries predicting worse HRQoL, greater disability, and lower return rates to productivity on the long term [132]. Worse physical functioning was reported in patients suffering from lower extremity injury compared to upper-extremity injury [39]. Especially articular injuries were impactful as opposed to shaft injuries alone [39]. More distal lesions (in feet or ankles) [73] and traumatic amputations of the lower extremity [39] were reported to be the most influential, and injuries around the knee joint were the primary cause of impairment in sports [70]. Patients with fractures below the knee joint reported worse HRQoL compared to patients with fractures above it [75]. Also, severely injured patients with foot injuries presented with worse outcomes in terms of HRQoL compared to patients without [72]; concomitant injuries to midfoot fractures (Chopart and/or Lisfranc injuries) were associated with worse long-term HRQoL [143]. Lower extremity injuries and orthopedic surgeries were independent predictors for daily pain and use of pain medication on the long-term [149]. Upper extremity injury was also found to be a predictor for worse HRQoL, more disability, and reduced return to work in some studies [31, 142, 156], especially when the brachial plexus was injured [71].
  Although some studies (*n* = 10) indicated no (significant) correlation between (the severity of) head and/or neurological injuries and HRQoL [29, 38, 46, 48, 63, 65, 67, 86, 138, 142], the majority (n = 16) of publications did [31, 36, 37, 42, 43, 48, 54, 55, 60, 64, 66, 68, 69, 74, 136, 155]; especially mental and cognitive function were affected as well as the pain domain [36, 43, 48, 54, 55, 64, 66, 69, 156], with an initial lower Glasgow Coma Scale (GCS) predicting worse HRQoL [60, 68, 69]. GCS appeared to be a reliable predictor of HRQoL in some populations [60, 68], and was negatively associated with self-reported physical health. Spinal cord injuries, paraplegia in patients, and higher modified Abbreviated Injury Scale (AIS) spine injuries resulted in poorer HRQoL [31, 39, 60, 136, 140, 155].
  Severe chest injury predicted reduced HRQoL scores after 1 month [30] and frequent use of pain medication one-year post-injury [149]. Flail chest in patients with multiple rib fractures was not a predictor for poor HRQoL [141]. In patients with a flail chest, a concomitant sternum fracture was a significant predictor for worse HRQoL [53].
  Two studies found patients with injuries to the abdomen to be less likely to report poor HRQoL [35, 142]. In addition, no significant association was found between HRQoL and pelvic fractures managed with ORIF [138], not even with presence of acetabular, genitourinary or neurological injury [38]. Patients with pelvic ring fractures stabilized with ORIF scored worse HRQoL scores when concomitant lower extremity injuries were present [131]. Facial injuries and fractures predicted worse HRQoL and more cognitive limitations [60, 155].
  The number of body regions injured was significantly associated with long-term HRQoL [133, 159].
II. **Injury severity**: 33 studies [29, 30, 33, 34, 36–40, 42–44, 46, 49, 52, 54, 56–61, 129, 130, 133–135, 139, 142, 147, 150, 154, 155] reported on the relationship between injury severity (ISS/AIS) and HRQoL. 21 of those studies [29, 30, 33, 34, 36, 37, 44, 52, 54, 56, 59, 61, 129, 133–135, 139, 142, 150, 154, 155] demonstrated that injury severity was a predictor for worse HRQoL or slower improvements in HRQoL scores over time. Patients with higher ISS also showed a reduced capacity to work and reported more pain two years after trauma [54, 142, 155]; the latter was also associated with AIS-5 lesions [54]. Patients’ perceived injury severity correlated significantly with HRQoL, with higher scores resulting in lower 6-month scores on both the physical and mental domains [33].
III. **Type of trauma and mechanism of injury**: In one study, blunt trauma predicted poor HRQoL 90 days after injury [35]. Other mechanisms of injuries that were associated with HRQoL deficits included pedestrian struck mechanisms [133, 142], self-inflicted injuries [137], violent mechanisms, such as assault [142, 157, 158], work-related injuries [149], falls [149], and firearm injuries in comparison to cutting or piercing penetrating traumas [158].
IV. **Characteristics of diagnoses and physiological parameters**: The number of diagnoses had no significant effect on HRQoL or disablement [46, 77]. According to Lichtveld et al. [78], HRQoL is not influenced by the fact that diagnoses are missed initially during prehospital care.
  Tachycardia and hyperglycaemia in poly-trauma patients sustaining spine injuries were early predictive parameters for worse HRQoL [140].
V. **Complications**: No association between Mental Component Score (MCS) or Physical Component Score (PCS) and complications, such as Acute Respiratory Distress Syndrome (ARDS), pulmonary embolism, and sepsis was found [30].
VI. **Pain**: Presence of pain and pain intensity were presented as predictors for worse HRQoL [67, 80, 81]. Longer-term pain severity (a HRQoL subscale in the SF-36, EQ-5D and Trauma Outcome Profile, or TOP) was associated with pre-trauma pain level [54].

#### Hospital-related parameters

I. **Intensive Care Unit (ICU) stay**: ICU admission was associated with poor HRQoL in some studies [139, 145, 159]. Furthermore, the number of failing organs during ICU stay was an independent predictor for poor HRQoL [52], as well as duration of ICU admission and treatment [35, 152].
  The Acute Physiology And Chronic Health Evaluation II (APACHE-II) is a classification used by ICUs to measure disease severity. An APACHE-II < 10 predicted better HRQoL for poly-traumatized patients after one and two years [29]. The Simplified Acute Physiology Score II (SAPS II) is another method to measure disease severity in the ICU and was significantly correlated with various HRQoL subscales [34].
  Intubation upon arrival in the Emergency Room (ER) was an independent determinant for poor HRQoL [52]. Length of ventilator treatment was a significant predictor for several HRQoL subscales [32, 48].
II. **Length of hospital stay**: Longer duration of hospital and/or rehabilitation stay was significantly associated with reduced HRQoL [2, 68, 145, 159], less return to work [32, 40], and higher frequency of daily pain and use of pain medication on the long-term [149]. One study [46] found no significant effect on disablement.
III. **Characteristics of hospital and rehabilitation care**: Patients receiving surgery during primary hospitalization reported reduced HRQoL [2]. A long duration of medical care [41, 58] or an inadequate treatment according to the patient [41] had a negative impact on HRQoL. Satisfaction with hospital stay seemed to have a positive effect on several HRQoL subscales [44]. One study, however, indicates no significant difference between patients who were satisfied and who were not [79].
  Regarding rehabilitation, satisfaction was a significant determinant of HRQoL [48], whereas discharge destination was not [30].
  No difference between level one and level two trauma centers regarding HRQoL was found [57].

#### Other parameters

I. **Coping**: Mental wellbeing improved when approach-oriented coping was increased [67]. Emotional coping and resilience predicted better HRQoL [81].
II. **Compensation and insurance**: No association between MCS or PCS and American Medicare insurance was found [30]. Patients who did not receive any workers’ compensation reported better HRQoL than patients who did [83]. Having made a (unsettled) compensation claim and using a lawyer were associated with poor HRQoL [135].
III. **Disability**: Disability was a significant predictor for worse HRQoL [30, 37, 43, 48, 58, 81], especially for physical function-indicators. There was a strong correlation between HRQoL and the global level of functioning: functional (in)dependence had a great impact on HRQoL; more independent patients reported better HRQoL [85].
  Pre-injury disability and poor preadmission HRQoL were associated with lower HRQoL [29, 158]. Frailty in patients was reported to be a useful predictor of poor HRQoL in trauma patients [153].
IV. **Cognitive behavioral therapy (CBT)**: CBT had an impact on depression and anxiety following poly-trauma; however, early CBT didn’t seem to significantly affect other domains of HRQoL [62].

## Discussion

This review aimed to provide a comprehensive overview of the parameters that influence the health-related quality of life of patients after severe trauma.

### Summary of main results

In this review, 90 studies presenting 23 parameters were discussed. Among patients suffering severe trauma, several factors were associated with HRQoL. Multiple parameters predicting reduced HRQoL came forward several times (in at least more than three studies), namely higher age [2, 29, 31, 33, 35–37, 41, 42, 44, 46, 133, 134, 136, 139, 146–150, 152, 154, 155, 158], the female gender [2, 34–36, 41, 48, 50–52, 68, 133, 139, 141, 150, 151, 158], body region of injury [29–31, 34–39, 42–44, 46, 48, 53–55, 60, 63–76, 86, 131–133, 136, 138, 140–143, 149, 155, 156, 159] (especially lower extremity injuries [29–31, 35, 39, 42, 44, 70, 72–75, 131, 132, 142, 143, 149, 155]), higher rate of injury severity (as indicated by the ISS) [29, 30, 33, 34, 36, 37, 44, 52, 54, 56, 59, 61, 129, 133–135, 139, 142, 150, 154, 155], lower achieved educational level [31, 32, 35, 37, 40, 41, 54, 68, 149], presence of mental/psychiatric disorders [30, 33, 37, 42, 49, 63, 67, 76, 84, 86, 130, 142, 144, 145, 149] or other comorbidities [2, 31, 36, 41, 55, 139, 140, 149, 152, 155, 158], a longer duration of hospital stay [32, 40, 68, 138, 145, 149, 159], and a high level of disability [29, 30, 37, 43, 48, 58, 81, 85, 153, 158].

### Strengths and weaknesses of the review

Although this review attempted to shed light onto the parameters that predict HRQoL in patients suffering severe trauma in a comprehensive and systematic way, it is not without limitations.

We acknowledge that methodological quality of research is important in studies investigating the predictive value of certain variables. However, since results of its evaluation would not have been further utilized, the risk of bias has not been assessed in this review. The different levels of reliability and hence relevance of the publications could therefore not be taken into account. We consider this a limitation of our review, since some of the included studies are of lesser methodological quality, e.g., due to either a retrospective nature of analysis, a low number of included patients, or other methodological flaws. These studies, however, serve themselves well for identification of predictors.

Another limitation is the fact that no meta-analysis was performed, considering the supposed heterogeneity among the included studies. The studies differed in tools used to assess (HR)QoL, characteristics of study populations, and study focus. Pooling of results was hence impossible and would not be valuable. Our narrative manner of discussing predictors may be inferior to the quantitative summarization of the results.

Finally, it should be explicitly noted that the results analyzed in this review represent outcomes for a population ill-defined in literature. The average ISS of the populations analyzed in this review ranged from 9 to 57. Although poly-trauma patients are conventionally identified by an ISS threshold of 15 [87–92], many definitions are adopted and used in literature, as illustrated by the included studies in this review as well. Therefore, for this review, we chose not to use a certain ISS cut-off value as an eligibility criterion for inclusion, as for populations of patients categorized as having suffered ‘severe’ or ‘major’ injury, multiple definitions and thresholds are used in literature, making the use of one hard cut-off value not accurate enough. Moreover, these terms are broadly used in scientific literature, contributing valuably to every review; exclusion of those articles would result in a huge gap from a methodological point of view and would affect the comprehensiveness of this review. Given the fact that ISS thresholds have been chosen arbitrarily [19, 20], and several publications have demonstrated that the poly-trauma ISS threshold excludes significantly morbid trauma patients [93–95], in whom (predictors of) patient-reported outcomes (PROs), such as HRQoL, are of more importance, we regard inclusion of patients with lesser ISS as a limitation of lesser relevance. After all, ‘poly-trauma patients’ were not necessarily the group of patients that formed the focus of this review; we mainly regard the ill-defined concepts of ‘severe’ and ‘major’ trauma in literature as a limitation of this review.

Despite these limitations, we nevertheless believe it is a major strength that this study includes a comprehensive review of the HRQoL predictors of severely injured patients throughout the years, with inclusion of studies ranging from 1994 to 2022, the majority of which was conducted prospectively. Included studies were not limited to the publications written in the English language, nor injury-specific populations, resulting in a smaller chance of missing relevant studies. Also, application of eligibility criteria and data-extraction were performed in duplicate, reducing the risk of errors.

### Implications for clinical practice and future research

The abovementioned findings might be of value to health-care givers, for them to recognize that some incoming patients with a certain profile (e.g., post-menopausal women, patients suffering from lower extremity injuries) might need more attention in their rehabilitation process and (post-)hospital care, in comparison to other patient groups (e.g., young, male patients without pre-existing diseases that are able to return to work quickly) of whom is to be expected that HRQoL won’t be as affected as the former patient group.

HRQoL is not only a concern for clinical practitioners, but also for researchers and policy makers [96, 97]. We believe that research and political priorities need to align with patient needs and wishes. With the information provided in this review, we therefore recommend research on the following topics:

I. Interestingly, a difference in HRQoL was found between men and women. Previous studies [98–105] have readily demonstrated this gender-related difference for other outcomes, such as survival and complication rate, explained by some by immunological and hormonal differences [106–114]. More research on whether this applies to HRQoL as well, might facilitate personalized medicine.
  Moreover, we found only few studies investigating the parameters predicting HRQoL in severely injured children [45, 133, 136, 159]. Since this is valuable information in this population just as it is in adults, further research on this topic is desired [115].
II. It is hypothesized that missing injuries during the initial surveys, a known phenomenon in care of severely injured patients (several studies indicate that higher ISS contribute to the incidence of missing injuries [116–120]), may have a life-long impact on the HRQoL. More research is needed to assess the effects of the tertiary survey on HRQoL, which has proven to be beneficial to other outcomes [121].
III. As mentioned before, the definitions and thresholds used in literature for severely injured patients, appear to be chosen arbitrarily. The ISS threshold of 16 has proven to correlate well with mostly mortality-related outcomes [20, 122–128], but whether this cut-off is discriminative enough for other outcomes of interest, such as HRQoL, has not yet been demonstrated. Therefore, we encourage more research on the ISS and other trauma severity indices to help distinguish ‘severe’ from ‘mild’ or ‘moderate’ injury, and ‘major’ from ‘minor’ trauma. This will help resolve vagueness regarding the various definitions present in literature, and will prevent semantic discussions concerning severity of injuries, benefitting scientific research.
IV. Further studies are needed regarding the patient-specific care that will benefit HRQoL of severely injured patients the most. Many of the identified parameters violating satisfactory HRQoL may be targeted in clinical practice, including pain management, hospital satisfaction, and mental health management. Although some variables are unchangeable, given determinants, such as age, gender, or injured body region, they still may add value to current information provision and patient approach, hence improving overall patient care and possibly HRQoL.
  Speaking of the latter type of factors, surprisingly, we found no literature on the effects of race and ethnicity on HRQoL—in light of the current increasing focus on Diversity, Equity and Inclusion (DEI) in healthcare, this might be worthy to further research.

Hopefully, information provided in this review will provide impetus for future research that is needed to provide evidence on how certain patients presenting with certain predictors for worse HRQoL should be approached and cared for after severe trauma, e.g., by means of (tailor-made) interventional and recovery programs.

## Conclusion

Age, gender, body region, and severity of injury were found to be good predictors of health-related quality of life in patients suffering severe trauma. We believe that these indicators might be crucial for identifying those patients that are in need of more personalized care. They may even aid in decreasing or preventing adverse post-traumatic consequences, hence improving HRQoL. A patient-centered approach, based on individual, demographic, and disease-specific predictors, is therefore highly recommended.

### Supplementary Information

Below is the link to the electronic supplementary material.

Supplementary file1 (DOCX 82 KB)

## Data Availability

The authors declare that the data supporting the findings of this study are available within the paper, and its supplementary information files (10.1007/s00068-023-02276-y).
