# Supplementary material for: Parameters influencing health-related quality of life after severe trauma: a systematic review (part II)
Source: Eur J Trauma Emerg Surg. 2023 May 15;50(1):93–106. doi: 10.1007/s00068-023-02276-y (PMC10923745; doi:10.1007/s00068-023-02276-y)
Supplement: Supplementary file 1 — Supplementary file1 (DOCX 82 KB) [file 68_2023_2276_MOESM1_ESM.docx]

**Appendices A and B: Supplementary data**

The following are the supplementary data to this article:

Appendix A. Conducted searches in Cochrane Library, EMBASE, PubMed, and Web of Science.

Appendix B. Table 1 – Studies included in review: population characteristics and study findings.

**Appendix A: Conducted searches in Cochrane Library, EMBASE, PubMed, and Web of Science**

**Search in Cochrane Library:**

#1 MeSH descriptor: [multiple trauma] this term only

#2 multiple trauma

#3 polytrauma

#4 polytraumas

#5 multiple injury

#6 multiple injuries

#7 multiple wounds

#8 major trauma

#9 severe trauma

#10 severe injury

#11 severe injuries

#12 multitrauma*

#13 MeSH descriptor: [Injury Severity Score] this term only

#14 ISS

#15 Injury Severity Score

#16 ISS score

#17 ISS-score

#18 MeSH descriptor: [patient outcome assessment] this term only

#19 Outcome

#20 Patient outcome assessment

#21 Assessments, Patient Outcome

#22 Patient Outcome Assessments

#23 Patient-Centered Outcomes Research

#24 Patient Centered Outcomes Research

#25 Patient Outcomes Assessment

#26 MeSH descriptor: [Quality of life] this term only

#27 quality of life

#28 QoL

#29 Life Quality

#30 Health-Related Quality Of Life

#31 HRQOL

#32 MeSH descriptor: [Patient Reported Outcome Measures] this term only

#33 Patient Reported Outcome* Measures

#34 Patient-Reported Outcome Measures

#35 PROM

#36 patient-reported outcome

#37 PRO

#38 PROMS

#39 patient reported outcome

#40 burden

#41 health-related burden

#42 (#1 OR #2 OR #3 OR #4 OR #5 OR #6 OR #7 OR #8 OR #9 OR #10 OR #11 OR #12) AND (#13 OR #14 OR #15 OR #16 OR #17) AND (#18 OR #19 OR #20 OR #21 OR #22 OR #23 OR #24 OR #25 OR #26 OR #27 OR #28 OR #29 OR #30 OR #31 OR #32 OR #33 OR #34 OR #35 OR #36 OR #37 OR #38 OR #39 OR #40 OR #41)

**Search in EMBASE:**

1 (multiple trauma OR major trauma OR polytrauma OR polytraumas OR multiple injury OR multiple injuries OR Multiple wounds OR severe trauma OR severe injury OR severe injuries OR multitrauma*).af

2 (Injury Severity Score OR ISS OR Injury Severity Score OR ISS score OR ISS-score).af

3 (Outcome OR Patient outcome assessment OR patient outcome assessment OR Assessments, Patient Outcome OR Patient Outcome Assessments OR Patient-Centered Outcomes Research OR Patient Centered Outcomes Research OR Patient Outcomes Assessment OR quality of life OR quality of life OR QoL OR Life Quality OR Health-Related Quality Of Life OR Health Related Quality Of Life OR HRQOL OR HRQoL OR Patient Reported Outcome Measures OR Patient Reported Outcome* Measures OR Patient-Reported Outcome Measures OR PROM OR patient-reported outcome OR PRO OR PROMS OR patient reported outcome OR burden OR health-related burden)

4 1 and 2 and 3

**Search in PubMed:**

((multiple trauma[MeSH] OR multiple trauma[tiab] OR polytrauma[tiab] OR polytraumas[tiab] OR multiple injury[tiab] OR multiple injuries[tiab] OR Multiple wounds[tiab] OR multitrauma*[tiab] OR major trauma[tiab] OR severe trauma[tiab] OR severe injury[tiab] OR severe injuries[tiab]) AND (Injury Severity Score[Mesh] OR ISS[tiab] OR Injury Severity Score[tiab] OR ISS score[tiab] OR ISS-score[tiab]) AND (Outcome[tiab] OR Patient outcome assessment[tiab] OR patient outcome assessment[MeSH] OR Assessments, Patient Outcome[tiab] OR Patient Outcome Assessments[tiab] OR Patient-Centered Outcomes Research[tiab] OR Patient Centered Outcomes Research[tiab] Patient Outcomes Assessment[tiab] OR quality of life[MeSH] OR quality of life[tiab] OR QoL[tiab] OR Life Quality[tiab] OR Health-Related Quality Of Life[tiab] OR Health Related Quality Of Life[tiab] OR HRQOL[tiab] OR HRQoL[tiab] OR Patient Reported Outcome Measures[MeSH] OR Patient Reported Outcome* Measures[tiab] OR Patient-Reported Outcome Measures[tiab] OR PROM[tiab] OR patient-reported outcome[tiab] OR PRO[tiab] OR PROMS[tiab] OR patient reported outcome[tiab] OR burden OR health-related burden))

**Search in Web of Science:**

((multiple trauma[MeSH] OR multiple trauma[tiab] OR polytrauma[tiab] OR polytraumas[tiab] OR multiple injury[tiab] OR multiple injuries[tiab] OR Multiple wounds[tiab] OR multitrauma*[tiab] OR major trauma[tiab] OR severe trauma[tiab] OR severe injury[tiab] OR severe injuries[tiab]) AND (Injury Severity Score[Mesh] OR ISS[tiab] OR Injury Severity Score[tiab] OR ISS score[tiab] OR ISS-score[tiab]) AND (Outcome[tiab] OR Patient outcome assessment[tiab] OR patient outcome assessment[MeSH] OR Assessments, Patient Outcome[tiab] OR Patient Outcome Assessments[tiab] OR Patient-Centered Outcomes Research[tiab] OR Patient Centered Outcomes Research[tiab] Patient Outcomes Assessment[tiab] OR quality of life[MeSH] OR quality of life[tiab] OR QoL[tiab] OR Life Quality[tiab] OR Health-Related Quality Of Life[tiab] OR Health Related Quality Of Life[tiab] OR HRQOL[tiab] OR HRQoL[tiab] OR Patient Reported Outcome Measures[MeSH] OR Patient Reported Outcome* Measures[tiab] OR Patient-Reported Outcome Measures[tiab] OR PROM[tiab] OR patient-reported outcome[tiab] OR PRO[tiab] OR PROMS[tiab] OR patient reported outcome[tiab] OR burden OR health-related burden))

**Appendix B: Table 1 – Studies included in review: population characteristics and study findings**

***Table 1 – Studies included in review: population characteristics and study findings***

| **Author [reference], year, study design, country** | **Study population, ISS, age (years), men %** | **Comparison group** | **(HR)QoL assessment instrument** | **Time point(s) of outcome assessment** | **Predictors/variables associated with HRQoL** |
| --- | --- | --- | --- | --- | --- |
| Holbrook [131], 1994, prospective cohort study, the USA | Patients admitted with major trauma (n = 42), mean ISS 15 (SD 10.1, range 5-42), mean age 30 (SD 13.1, range 18-69), 74% |  | QWB-scale | 3 months post-trauma | - Predischarge depression score is significantly and independently predictive of outcome - Injury severity and body region do not appear to be associated with functional limitation |
| Gruen [132], 1995, retrospective cohort study, the USA | Patients with pelvic ring fractures requiring ORIF of pelvis (n = 48), average ISS 23.8 (SD 10.9), mean age 34.9 (SD 14.9), 64.6% |  | SIP | 1-year post-trauma | - Concomitant lower extremity fractures reported worse HRQoL (higher mean SIP individual scores in ambulation-domain (17.9 ± 19.1)) compared with patients without lower extremity injuries (7.8 ± 9.4). - Unstable pelvic ring fractures managed with ORIF had mild disability. |
| Vazquez Mata [29], 1996, prospective cohort study, Spain | Polytraumatized patients admitted to ICU, mean ISS 23.6 ± 0.6, age 31.15 ± 0.86, 79,8% | Non-polytraumatized patients | Quality-of-Life-questionnaire | Admission, 1 and 2 years | Predictors for worse reported HRQoL:   - Older age ≥ 60 yrs - Worse pre-admission HRQoL scores - APACHE-II ≥ 10 - ISS ≥ 25 - Presence of long bone fractures   Severe head injury was not a predictor for poor HRQoL. |
| Van der Sluis [45], 1997, retrospective cohort study, the Netherlands | Paediatric patients (< 16 years) surviving polytrauma (n = 50) (the majority [74%] of which caused by traffic accidents), ISS 29 (range 17-57), age 11 (6-15), 50% | Healthy Dutch sample of 18- to 24-year-olds | RAND-36 | 7 to 11 years after injury (9 years on average) | - Paediatric patients reported no significant difference regarding physical and emotional HRQoL with the reference population |
| Fern [133], 1998, retrospective cohort study, Canada | Patient cohort with multiple extremity injuries (MEI; n = 54), mean ISS MEI-cohort 16.2 (SD 2.5), mean ISS control group 17.4 (SD 2.9), mean age MEI-cohort 39.3 (SD 4.0), mean age control group 43.9 (SD 8.5) | Control group with major injuries not focused on extremities (TC) and US population normative data. | SF-36 | 2 years post-injury | Patients with multiple extremity injuries (MEI) tend to demonstrate a trend towards longer-term disability compared to trauma patients without MEI. |
| Ruchholtz [63], 1999, prospective cohort study, Germany | Patients (n = 33) surviving suicide attempt (the majority [75%] by means of leaps from great height), mean ISS 40 ± 15, age 38 ± 15, 57% | Patients with good or satisfactory outcomes (defined as the combination of a GAF grading distinctly above 51 points and a subjective 1 to 3 scoring in quality of life) were compared with a cohort of patients with adverse outcomes (defined as indefinite psychiatric rehabilitational care, a GAF scale <50 points, and a QoL of 4-6 on the subjective scale) in order to identify predictors. | Subjective scale | 6.1 ± 3 years after trauma (range 16 months - 13 years) | Adverse HRQoL outcomes were more often seen in:   - Patients with chronic affective and schizophrenic disorders - Severe TBI (GCS < 9) (however, no significant association was reported) |
| Turchin [72], 1999, prospective cohort study, Canada | Patients (n = 28) with foot injuries (calcaneal and/or talar fracture(s), and/or Lisfranc fracture dislocations, other midfoot fractures, metatarsal fractures, phalangeal fractures). Mean ISS 25 (range 13-41), mean age 34 (range 18-63 years), 60.7% | Multiply injured patients without foot injuries were matched in a blinded fashion | SF-36, WOMAC | Mean duration follow-up 62 months | Worse total SF-36 scores were reported in patients with foot injuries compared to patients without foot injuries. Specifically, the following domains were affected:   - Physical functioning - Role physical - Bodily pain - Social functioning - Role emotional |
| Anke [82], 2003, retrospective cohort study, Sweden | Major trauma patients (n = 69), mean ISS 25 (range 17-50), median age 26 (12-72), 70% | Follow-up scores were compared to situation as it was prior to trauma | LiSat-9 | 3 years after trauma | The following factors have a significant impact on satisfaction with life as a whole:   - A strong sense of coherence - A sufficient social network quality   The following factor is related to low satisfaction with life as a whole, satisfaction with partner relations, and family life:   - Lower level of cognitive function |
| DePalma [58], 2003, prospective cohort study, the USA | Patients (n = 64) surviving major trauma, who are treated in and discharged from a level 1 trauma centre and admitted with an ISS >24, mean ISS 34.0 (SD 11, range 25-75), age 47.7 years (25-75), 68.8%. | Follow-up scores were compared to situation as it was prior to trauma | SIP, subjective scale | 6 months to 24 years post-injury | - ISS not significantly correlated with total SIP score or SIP physiological / psychological - On the subjective scale, gender did not affect the HRQoL significantly - On the subjective scale, the length of needed medical care was significantly associated with HRQoL - Patients with higher levels of disability rated lower HRQoL at time of interview |
| Dimopoulou [56], 2004, prospective cohort study, Greece | Patients surviving polytrauma (n = 87), which was mainly caused by car or motor vehicle collision, median ISS 22 (range 11-41), age 31 (16-85), 85.1% |  | NHP | 1 year after ICU discharge | - Injury severity according to ISS: an increase of ISS by 1 unit corresponds to a 12% increase in odds for having a score different than 0 in at least one dimension of the NHP part 1. |
| Holbrook [50], 2004, prospective cohort study, the USA | Patients (n =237) enrolled in the Trauma Recovery Project (both ISS <16 and ISS >15. Only patients >17 years were eligible for inclusion. 71.7% was male. | Comparisons by gender were made | QWB-scale | At discharge, and 6, 12, 18 months after discharge | - Female gender predicted poorer HRQoL than male gender |
| Pajonk [86], 2005, prospective cohort study, Germany | Patients (n = 65) with multiple injuries due to suicidal intentions (mainly [75%] by means of a leap from considerable height), mean ISS 39 ± 15, age 38 ± 16, 87% | Patients with good or satisfactory outcomes (defined as the combination of a GAF grading distinctly above 51 points and a subjective 1 to 3 scoring in quality of life) were compared with a cohort of patients with adverse outcomes (defined as indefinite psychiatric rehabilitational care, a GAF scale <50 points, and a QoL of 4-6 on the subjective scale) in order to identify predictors. | Subjective scale | 6.1 ± 2.9 years after trauma | The following factors predict better HRQoL in polytrauma patients:   - Full time or part time employment - Absence of psychiatric treatment - Absence of schizophrenic disorder   Persisting sequelae of severe TBI associated with adverse outcome. |
| Zelle [75], 2005, retrospective cohort study, Germany | Polytraumatized patients (definition of polytrauma did not include an ISS-threshold with injuries below the knee-joint (n = 389), average ISS 20.2 ± 9.3, age 25.4 ± 11.7, 73.8% | Patients with fractures above the knee joint were compared to patients with fractures below the knee joint | SF-12, HASPOC | Average 17.3 ± 4.8 years | Patients with fractures below the knee joint present with significantly higher HASPOC-subjective, HASPOC objective and total, and significantly lower SF-12 PCS (but not MCS) than patients with fractures above the knee joint. |
| Zelle [83], 2005, retrospective cohort study, Germany | Polytraumatized patients (definition of polytrauma did not include an ISS-threshold, both work-related and non-work-related (n = 637), average ISS 20.7 ± 9.7, age 26.4 (3-60), 75.4% | Patients receiving workers’ compensation were compared to patients who didn’t receive workers’ compensation | SF-12, HASPOC | 17.5 years (range 10–28 years | Patients receiving workers’ compensation (compared to NWC patients):   - Make use of medical aids and devices more often - Are more often retired due to injury - Are more likely to get enrolled in patient rehabilitation after injury - Spend significantly longer time at rehabilitation - Inferior outcomes as measured by HASPOC-subjective/HASPOC-objective/HASPOC-total/PCS of SF-12 (but not MCS SF-12).   Psychological factors such as secondary gain from illness contributed to the inferior outcomes in workers’ compensation patients. |
| Kiely [30], 2006, prospective cohort study, the USA | Patients with non-neurologic blunt injury (n = 203), ISS 17.1, mean age 44.5, 66.5% | Comparisons were made with the different time points and with population norms | SF-36 | 1- and 6-months post-injury | A poor PCS at 1 month was predicted by the following factors:   - Presence of extremity injury (even if minor) - Presence of significant (severe) chest injury   A poor MCS at 1 month was predicted by the following factors:   - Race (lower scores in blacks)   A poor PCS at 6 months was predicted by the following factors:   - Lower FIM score after 1 month - Absence of significant head/neck injury   A poor MCS score at 6 months was predicted by the following factor:   - PTSD at 1 month   Not associated with either MCS or PCS were:   - Social support - Age - Complications - Insurance - Discharge destination |
| Lichtveld [78], 2006, prospective cohort study, the Netherlands | Polytraumatized patients (n = 335) in whom influence of missed injuries was investigated, median ISS 21 (16-75), age 37.6 (SD 20.4), 72.4% |  | SIP, EQ-5D, subjective scale | 12-18 months post-injury (mean: 15 months) | HRQoL is not affected by missed injuries during prehospital care |
| Post [46], 2006, retrospective cohort study, the Netherlands | Polytraumatized patients (n = 53) with a mean number of diagnoses of 5, mean ISS 23.5 (16-54), mean age 37.3 (19-64), 81.1% |  | SIP | Average follow-up 1.8 years (SD 0.3; range 1.3-2.2) | The following factors do not predict disablement:   - ISS - Length of hospital stay - Number of diagnoses   The following factors do predict disablement:   - Age (OR = 1.07)   No significant differences were found in SIP scores between patients with severe head injury (AIS head/neck-score ≥ 4) and those with mild to moderate head injury (AIS head/neck-score 1 – 3) or no head injury. With respect to the extremities no differences were found between patients with mild to moderate injury (AIS score 1 – 3) or no injury. |
| Holbrook [134], 2007, prospective cohort study, the USA | Adolescent patients with major trauma (n = 401), mean ISS 10.8 (SD 7.4), mean age 15.0 (SD 2.3), 71% | US norms of healthy adolescents | QWB-scale | 3, 6, 12, 18, 24 months post-discharge | Risk factors for HRQoL deficits were:   - Female sex - Age > 15 years - Perceived threat to life - Pedestrian struck mechanism - ISS of greater than 16 - Three or more body regions injured |
| Holtslag [31], 2007, prospective cohort study, the Netherlands | Patients surviving major trauma (n = 335), all with ISS >15, mean ISS 24.9 (SD 10.6), age 37.7 (SD: 17.1), 74.3% | Dutch population norm | EQ-5D | 451 days (SD: 47 days); range: 12-18 months | Reduced HRQoL was related to:   - Age: patients ≥ 55 years reported significantly lower EQ-VAS and EQ-US scores - Achieved educational level: patients with no further education beyond primary school have lower EQ-VAS and EQ-US scoring - BMI: patients with BMI < 25 have higher EQ-VAS scores than patients with a BMI ≥ 25 - Comorbidity: patients with comorbidity have lower EQ-VAS and EQ-US scores than patients without comorbidity - Spinal cord injury (SCI): patients with this injury have lower EQ-US scores than patients without SCI - Lower extremity injury (LEI): patients with this injury have lower EQ-US scores than patients without LEI - Upper extremity injury (UEI): patients with this injury have lower EQ-US and EQ-VAS scores than patients without UEI - Brain injury (TBI): patients with TBI have worse HRQoL than patients without TBI |
| Hładki [135], 2007, prospective cohort study, Poland | Multiply injured patients (n = 827), mean ISS 27.6, mean age 44.7 |  | The classification of disability by the International Classification of Impairment, Disabilities and Handicaps |  | Slowed improvements in HRQoL were associated with:   - Increase in severity of injuries - Advanced age of patients |
| Janssen [79], 2007, retrospective cohort study, Germany | Severely injured patients (n = 90) who were asked to identify determinants of satisfaction with regard to acute hospitalization, mean ISS 22.9 (SD 9.5), age 42.3 (SD 12.9), 74.4% | Patients satisfied with hospital care were compared to patients dissatisfied with hospital care | SF-36 | Mean 3.7 years (SD: 1.5) | Hospital care satisfaction did not determine HRQoL |
| Lippert-Grün [64], 2007, prospective cohort study, Germany | Patients (n = 49) with severe traumatic brain injury defined as a GCS < 9 for more than 24 hours with and without concomitant polytrauma, ISS 33.5, median age 32 (15-68) years, 77.6%. | Patients with isolated TBI were compared with patients with TBI and concomitant polytrauma | SF-36 | 6 and 12 months after trauma | TBI is a predictor for worse HRQoL post-injury as measured by SF-36 scoring, except for physical functioning 12 months post-injury. |
| Pirente [62], 2007, prospective (RCT) study, Germany | Patients after trauma to whom cognitive behavioural therapy was given on the surgical ward (n = 45), total mean ISS 22.4 (range 9-50), age 38 (18-69), 70.7%. | A control group to whom no cognitive behavioural therapy was given on the surgical ward (n = 47). | SF-36 | Discharge, 6- and 12-months post-injury | Early CBT has no effect on QoL of polytrauma patients. Only on the dimensions depression (at discharge/6 months/12 months) and anxiety (at discharge and 6 months) significant effects were found. |
| Soberg [32], 2007, prospective cohort study, Norway | Multiply injured patients (n = 100), mean ISS 28.1 (SD 11.3), age 34.5 (SD 13.5), 82% | Norwegian general population norms and different disease population norms | SF-36 | 6 weeks after discharge, 1- and 2-years post-injury | Not returning to work was associated with:   - Male gender - Length of ventilator treatment - Length of stay in hospital/rehabilitation institution > 20 weeks - Low social functioning   Returning to work was associated with:   - Higher education - White-collar work - Better social functioning - Shorter length of stay   Not returning to work was associated with worse SF-36 scores. |
| Giannoudis [47], 2008, retrospective cohort study, the UK | Patients having undergone open reduction and internal fixation (ORIF) to pubic symphysis (n =74), median ISS 25.5 (range 18-34), age 40.6 (16-75), 75.7% | UK population norms | EQ-5D | Mean follow-up 41.7 months (28-89 months) | HRQoL was poor in men of all age groups. |
| Harris [136], 2008, retrospective cohort study, Australia | Patients with major trauma (n = 355), mean ISS 24.3 (range 16-66), mean age 47.8 (range 19-91), 72.1% |  | SF-36 | Mean 41.0 months (12-74 months) | Better physical health associated with:   - Increasing time since injury - Lower ISS   Poor physical health associated with:   - Having a settled compensation claim - Having an unsettled compensation claim - Using a lawyer   Poor mental health associated with:   - Having an unsettled compensation claim |
| Holtslag [61], 2008, prospective cohort study, the Netherlands | Polytraumatized patients (n = 335), ISS > 15 (not further specified), 37.7 years (SD 17.1), 74.3% |  | EQ-5D | 15 months post-injury | Higher injury severity is correlated with reduced HRQoL. |
| Janssen [44], 2008, retrospective cohort study, Germany | Severely injured patients (n = 90), mean ISS 22.9 (SD 9.5; range 9-50), mean age 42.3 (SD 12.9; range 22-71), 74.5%. | German population norm | SF-36 | Mean 4 years post-injury | The following factors predict poor SF-36 scores for several subscales:   - Higher age - Lower SES - Living together with partner - Severity of trauma according to ISS - Extremity injuries   The following factor predict positive SF-36 scores for several subscales:   - Satisfaction with hospital stay |
| Ulvik [34], 2008, prospective cohort study, Norway | Multiply injured patients admitted to ICU (n = 210), median ISS 25 (4-54), mean age 39 ± 17 (18-83), 81.0% | Pre-trauma scores were compared to post-trauma scores | EQ-5D | 2-7 years post-trauma (median 4.0 years) | HRQoL is impacted negatively by the following factors:   - SAPS II (severity of illness in ICU) - ISS - Female gender - Absence of severe head injury   HRQoL is impacted positively by the following factor:   - Time since trauma   Age didn’t influence HRQoL significantly. |
| Janssens [137], 2009, prospective cohort study, the Netherlands | Severely injured children (< 16 years, n = 40), mean ISS 24.9 (SD 11.1), mean age 8.9 (SD 4.6), 63% | Dutch general child population group | PedsQoL, EQ-VAS | Mean 7.3 years (SD 0.7 years) | The lowest scores on the PedsQL and the EQ-VAS were seen in teenagers and in respondents with spinal cord and/or severe cerebral injury.  The HRQL of children 6–8 years after major trauma did not significantly differ from healthy peers when measured with the PedsQL (81.2). |
| Baranyi [84], 2010, retrospective cross-sectional, Austria | Polytraumatized patients (n = 52), mean ISS 33.1 (SD 12.6), age 37.6 (SD 14.2), 73.1% |  | SF-36 | 1-year follow-up | Patients with full PTSD showed lowest HRQOL, followed by patients with partial PTSD and patients without PTSD. |
| Bilén [138], 2010, prospective study, Sweden | Majorly injured patients (n = 211), mean ISS 16.7 (SD 5.5), mean age 52.9 (SD 20.3), 64.9% | General population norms | SF-36 | 12 months post-injury | Patient with self-inflicted injuries presented with worse HRQoL (except for physical function). |
| Brasel [33], 2010, prospective cohort study, the USA | Polytraumatized patients (n = 426), mean ISS 13.3 (SD 7.9; desired outcome was considered in patients with ISS>15 only), mean age 42.0 ±16.9, 70.9% |  | SF-36 | During hospitalization, at 6 months | Poor physical HRQoL during hospitalization is correlated with the following factors:   - Greater perceived injury severity - Higher ISS - Older age   Poor mental HRQoL during hospitalization is correlated with the following factors:   - Higher PTSD symptom severity - Older age |
| Pape [39], 2010, prospective cohort study, Germany | Patients with multiple non-neurological injuries admitted to level 1 trauma centre (n = 637), ISS was >16 in all patients (not further specified), age 26.5 (SD 12.4; range 3-58), 75.4% | Patients with lower extremity injuries were compared to patients without lower extremity injuries; shaft injuries were compared to articular injuries; 2+ articular injuries were compared to no articular injuries. | SF-12, HASPOC | More than 10 years follow-up | The following type of injuries present with lower HASPOC scores and SF-12:   - 2 or more articular injuries - Lower extremity injuries - Combination of shaft and articular injuries - Higher AIS spine score - Lower extremity amputation   The following factors are associated with more favourable HASPOC scores:   - Higher age - Higher ISS |
| Probst [51], 2010, prospective cohort study, Germany | Polytraumatized patients who were followed up >10 years post-trauma (n = 637), mean ISS 21 ± 9.8, age 26.3 ± 12.6, 75% | Male gender participants were compared to female gender participants | SF-12, HASPOC | 17.5 ± 4.9 years | Women report worse HRQoL psychological scores, higher rates of PTSD and psychological support, longer sick leave time and show longer duration of rehabilitation than men. |
| Steel [48], 2010, retrospective cohort study, the USA | Multiply injured patients (n = 620; all with an ISS >16), of whom 64% had suffered TBI, mean ISS 20.7 (SD 4.9), age 26 (3-58 years), 75.3% | Patients with TBI were compared to patients without TBI | SF-12 | >= 10 years follow-up | Patients with TBI score significantly lower in the psychological SF-12 subscale (mean: 49.4; SD: 10.1) when compared with those without TBI (mean: 51.3; SD: 9.1).  No significant difference was found between patients with multiple injuries with and without TBI regarding the SF-12 physical subscale.  The following factors predict worse PCS significantly:   - Female gender - Age at time of injury (young age) - Length of time on ventilation - Less satisfaction with rehabilitation - Greater disability   The following factors predict psychological functioning significantly:   - Female gender - Satisfaction with rehabilitation - Disability |
| Tuchner [49], 2010, retrospective cohort study, Israel | Patients (n = 35) surviving terrorist attacks (all injuries were caused by suicide bombings), mean ISS 27 (±14.2), age 32.2 (SD 13.8; range 14-74), 48.6% | Israeli population norms | SF-36 | Mean 2.1 years (1-3.5 years) | - PTSD severity is correlated with total SF-36 scores and SF-36 MCS - Returning to main occupation is correlated with higher SF-36 MCS score   Age, gender, ISS do not predict HRQoL significantly. |
| Christensen [35], 2011, prospective (RCT) study, 26 countries | Patients in whom the effect of recombinant-activated factor VIIa for treatment of severe trauma injury with refractory bleeding (compared to placebo), defined as bleeding after receiving 4 units of RBCs despite standard haemostatic interventions, was investigated (n = 347), median ISS 29 (IQR 22-41), the mean ISS was 31 (SD 11), mean age 39 (SD 14), 75% | Trial participant values were compared to general population values | POLO-chart (GOS, EQ-5D, SF-36, TOP) | 3 months post-injury | The following factors predicted poor HRQoL 90 days post-injury:   - Higher age - Female gender - Injury to extremity - Blunt trauma injury - ICU stay >3 days - Repeated nonadherence to transfusion guidelines - Inability to work postinjury   Patients presenting with the following factors were less likely to report poor HRQoL:   - Injuries to the abdomen - Patients employed pre-injury - Higher education (university degree/equivalent professional qualification) |
| Gross [54], 2011, prospective cohort study, Switzerland | Patients with polytrauma (n = 102), ISS 27.1 ± 8.0 (all included patients had an ISS >16), age 39.7 (SD 20.5), 75% | Post-injury QoL (domain: pain) was compared to pre-injury QoL (domain: pain) | EQ-5D, SF-36, TOP | Median 2.4 years after injury | Predictors for pain at least 2 years post-injury according to TOP are the following:   - TBI - TRISS - Blue-collar profession - Greater BMI - Increasingly AIS V lesions - Lower educational level - Presence of preinjury pain   Predictors for pain post-injury according to the SF-36:   - Lower educational level - Presence of pre-injury pain - Increasingly severe AIS V lesions   Predictors for more pain at long-term follow-up in all three measures are the following:   - Presence of pre-injury pain (measured with identical instrument as post-injury) - Lower pre-injury educational level |
| Overgaard [139], 2011, cross-sectional study, Denmark | Moderate to severe trauma patients (n = 322), median ISS 17 (range 9-75), median age 34 (range 15-89), 71% | Matched control group with non-trauma patients | SF-36 | median 7.4 (range 6-9) years | Poor SF-36 outcome was not associated with:   - Presence of lower extremity or pelvic fractures - Presence of neurologic injuries   Poor SF-36 was associated with:   - Receiving any kind of surgery during primary hospitalization in all four Physical Component domains plus Social Functioning - Hospital admission >5 days, with statistical significance only reached in domain of Bodily Pain.   Patients who were admitted to the ICU did not report lower SF-36 scores than patients without ICU stay. |
| Ringburg [140], 2011, prospective cohort, the Netherlands | Multiply injured patients (n = 246), median ISS 22 (IQR 17-29), median age 40 (IQR 23-57), 66% | Dutch general population norms | EQ-5D | 1-year post-trauma | Worse HRQoL was associated with:   - Female gender - One or more comorbidities - Patients with a higher age (55 years or older)   Better reported HRQoL was associated with:   - A household composition of more than one person.   Type of prehospital care has no influence on the reported quality of life in any of the EQ-5D domains. |
| Soberg [40], 2011, prospective cohort study, Norway | Patients with polytrauma (n = 75), mean ISS 27.4 (11.7), age 36.1 (13.6), 77.3% | Patients returning to work (RTW) were compared to patients not returning to home (NRTW), and comparisons with general populations were made | SF-36 | 6 weeks after return at home from hospital/primary rehabilitation, at 1 years, at 2 years, 5 years postinjury | Predictors for RTW after discharge:   - Time - Higher education - Better physical functioning   Predictors for RTW after 1-year post-injury:   - Time - Gender - Higher educational level - Lower ability of approach-oriented coping - Lower NISS - Physical and social functioning   Predictors for RTW after 2 years post-injury:   - Higher educational level - Lower ability of approach-oriented coping - Physical and cognitive functioning.   RTW after 5 years post-injury was achieved more often in patients with:   - A higher education - A white-collar job - Short length of stay in primary hospital/rehabilitation |
| Zeckey [65], 2011, prospective cohort study, Germany | Population in whom polytraumatized patients were divided in groups of patients with and without head injury (n = 250 in total; n = 125 and n = 125, respectively), ISS 20.0 ± 8.7 (all with an ISS >15), age 27.9 ± 13.9, 81.6%. | Patients with head injury were compared to patients without head injury; results were compared to general German population norms as well. | SF-12, HASPOC | >= 10 years post-injury | No difference in the two components of the SF-12 (PCS/MCS) was found between the two groups. When compared to the general German population, the PCS was reduced in both groups independently of the presence of head injury. |
| Attenberger [77], 2012, prospective cohort study, Switzerland | Polytrauma survivors (n = 117), mean ISS 27.45 (SD 8.19; range 17-45), mean age 39.62 (SD 20.50), 75.2% | Patients with reduced capacity to work (RCW) were compared to patients without reduced capacity to work (NRCW); postinjury QoL was compared to pre-injury QoL | TOP | 2.7 ± 0.9 years after injury | Multiple injuries have no effect on HRQoL. |
| Gross [66], 2012, prospective cohort study, Switzerland | Patients (n = 111) polytraumatized mainly by traffic accidents (70.3%), mean ISS 27.9 ± 8.2 (all patients had an ISS >15), age 39.5 ± 20.9, 75% | TBI patients were compared to NTBI patients; post-injury status was compared to pre-injury status | EQ-5D, SF-36, TOP, NHP | Mean 2.7 ± 0.9 years | - HRQoL was reduced in both TBI and NTBI patients, however, the TBI group had poorer scores than the NTBI groups with regard to the EQ-VAS/EQ-5D/MCS SF-36. - According to the TOP, TBI patients report worse scores in the following domains: depression, anxiety, social interaction, mental functioning, satisfaction. - Differences in long term outcome between survivors of TBI vs NTBI were best visible in significantly reduces of SF-36 MCS and cognitive dimensions of other instruments. |
| Soberg [68], 2012, prospective cohort study, Norway | Polytrauma patients (n = 105), mean NISS 34.6 ± 12.6 (range 17-66), age 35.3 ± 14.0 years, 83% | General population norms; comparisons between different time points of outcome assessment | SF-36 | After injury (baseline), return to home, 1 year, 2 years, 5 years | Predictors satisfactory physical health (PCS) after return home:   - Higher GCS - Less time in hospital/rehabilitation - Getting around - Participation in society   Predictors satisfactory physical health (PCS) after 1 year:   - Getting around - Participation in society - Understanding and communicating - Higher education   Predictors satisfactory physical health (PCS) after 2 years:   - Higher GCS - Getting around - Participation in society   Time from injury, categorized as the 4 time points of measurement, was a factor that had a decreasing impact on the recovery of physical health.  Predictors of poor MCS:   - Time points of measurement - Female ender - Education - WHODAS II cognitive functioning - Participation in society |
| Andruszkow [74], 2013, retrospective cohort study, Germany | Patients with polytrauma (n = 281), divided into groups of patients with upper extremity injuries without TBI (group I), patients with concomitant upper extremity injury and TBI (group II) and patients with TBI but without upper extremity injury (group III). ISS in group I 20.2 ± 8.6, group II 32.1 ± 6.4, group III 33.8 ± 10.6; age 30.9 ± 11.4, 75.4% | Group I, II and III were compared to each other | SF-12, HASPOC | Median 17.5 years (10-28 years) post-injury | The lowest mean Hannover Score for Polytrauma Outcome occurred in Group I (patients with extremity injuries without TBI) indicating best HRQoL out of all three groups.  Patients in group II and III required psychological support most often.  HRQoL PCS according to SF-12 didn’t differ between all three groups; neither did SF-12 MCS. |
| Forslund [37], 2013, prospective cohort study, Norway | Polytraumatized patients (n = 91) of whom the majority (64.8%) had suffered severe TBI, mean ISS 29.5 (SD 13.1; range 4-59), age 31.1 (SD 11.3), 77%. | PCS and MCS scores from 1- and 2-years follow-up were compared; scores were also compared to USA and Norwegian population norms | SF-36 | 1, 2 years follow-up | Predictors poor HRQoL:   - >31 years - Lower educational achievement - Unemployment at time of injury - Depression (BDI >12) - Substance use at time of injury - CT-Marshall scores - Higher ISS - Severity of TBI - Functioning at 1 year according to FIM/CIQ/BDI scores - Changes in PCS/MCS scores between 1 and 2 years - Level of community integration |
| Simmel [41], 2013, retrospective cohort study, Germany | Polytraumatized patients with an ISS ≥ 25 were included (n = 127), average ISS 35.6 (36-75), average age 36 years, 76%. | German population norms (Bundesgesund-heitssurvey 1998) | EQ-5D, SF-36, TOP | 70 months (38-108 months) | HRQoL is negatively affected by the following factors:   - Higher age - Female gender - Low education - Pre-traumatic diseases - Difficulties with authorities/institutes - Unemployment due to the trauma - Long duration of treatment - Subjectively inadequate care/treatment in hospital |
| Von Rüden [73], 2013, retrospective cohort study, Germany | All polytraumatized patients with an ISS ≥50 were included (n = 88), average ISS 56.8, age 40 ± 17 years (range 18-63), 77.3% | Patients with an ISS ≥50 were compared to patients with an ISS < 50 | POLO-chart (GOS, EQ-5D, SF-36, TOP) | 3.6 years after trauma (range 18-78 months) | The more distal the lesions were located (foot/ankle) the more functional disability affected daily life. |
| Rainer [141], 2014, prospective cohort study, China | Moderate to major trauma survivors (n = 177), 61.6% of population ISS ≥ 16, mean age 52.9 (SD 19.9), 70.6% | Hong Kong population norms | SF-36 | 12 months after injury | HRQoL was significantly associated with:   - Age > 65 years (OR 4.77) - Male gender (OR 0.44) - Pre-injury health problems (OR 2.30) - Admission to ICU (OR 2.15) - ISS 26-40 (OR 3.72) |
| Tee [142], 2014, retrospective cohort study, Australia | Spine polytrauma patients (n = 479), median 14 (IQR 9-22), mean age 47.5 (SD 19.1), 71.8% |  | SF-12 | 1-year post-trauma | Early predictive factors of suboptimal physical HRQoL:   - Tachycardia (OR 1.88 [CI 1.11-3.19]) - Hyperglycaemia (OR 2.65 [CI 1.51-4.65]) - Multiple chronic comorbidities (OR 2.98 [CI 1.68-5.26]) - Thoracic spine injuries (OR 1.54 [CI 1.01-2.37]) |
| Marasco [143], 2015, retrospective cohort study, Australia | Major trauma patients with multiple fractured ribs (n = 397), mean ISS 22.5 (SD 11.8), mean age 53.9 (SD 18.8), 75.1% | Australian population norms | SF-12 | 6, 12 and 24 months | Better pain and SF-12 MCS scores were found in:   - Male patients - Older patients (> 55 years)   Presence of flail chest did not result in differences in outcome. |
| Scholten [36], 2015, prospective cohort study, the Netherlands | Patients with (mild/moderate/severe) TBI (n = 199), of whom the results of only moderate (GCS 9-12) and severe (GCS <9) patients were investigated due to eligibility criteria (median ISS 20.5 (10-29) and 29 (20-38), respectively). Age 43 (22.5-61.3), 64.8%. | Dutch population norms | SF-36, subjective QoL | 6- and 12-months post-trauma | The following factors were associated with reduced HRQoL after 6 and 12 months:   - Higher ISS - Female gender - Older age - Co-morbidity |
| Soberg [67], 2015, prospective cohort study, Norway | Patients (n =58) who have sustained severe injuries, mean NISS 33.7 (SD 13.0), age 37.8 (SD 14.7), 74% | General population norms | SF-36 | Baseline, 1, 2, 5, 10 years after injury | Significant predictors of PCS were the following:   - Physical functioning at 1 year - Cognitive functioning at 1 year - Bodily pain at 2 years - Change in coping.   Significant predictors of MCS at 10 years were the following:   - Increase in approach-oriented coping at 1 year - Vitality at 1 year - Social functioning at 2 years - Mental health at 2 years |
| Dinh [144], 2016, prospective cohort study, Australia | Patients with major trauma (n = 222), median ISS 9 (IQR 4-17), mean age 46.2 (SD 20.0), 77.7% |  | SF-12, EQ-5D | 3 and 6 months | Predictors of decreased PCS were:   - Presence of any lower limb injury - Increasing ISS   Predictors of decreased MCS were:   - Mechanism-related (assault, pedestrian) - Previous history of mental health diagnosis.   Increased PCS score between 3 and 6 months were associated with:   - Presence of abdominal injury or head injuries   Reduced odds of return to work were associated with:   - Increasing ISS (OR 98, 95% CI 0.97-0.99) - Upper limb injuries (OR 0.20, 95% CI 0.07-0.57) |
| Kaske [80], 2016, prospective cohort study, Germany | Trauma patients suffering from persisting pain even years after injury (n = 207), mean ISS 19.1 (SD 1.9), age 44 ± 18 years, 67% | Patients with a high level of pain (VAS >=5) were compared with patients presenting with less pain (VAS < 5) | TOP | 22.6 ± 3.8 months | Patients with severe pain presented significantly more restrictions of HRQoL in all dimensions of the TOP.  In patients with a VAS <5 9/10 dimensions were considered ‘normal’; only mental function was reduced. |
| Zibung, [60], 2016, rertrospective cohort study, Sweden | Bicycle trauma patients who attended a level I trauma centre (n = 148), only the results of patients with an ISS > 15 (n = 22; not further specified) were used, 48.5 (15-85), 63.5% |  | Hadorn's Quality of Life and Health questionnaire | 6 months post-crash | An ISS >15 is a predictor for impaired HRQoL. |
| Zwingmann [42], 2016, retrospective cohort study, Germany | Polytraumatized patients (n = 147), average ISS 28 ± 11, age 40.1 (SD 18.6), 75.5% | Age- and sex-adjusted values from a representative-norm German population. | EQ-5D, SF-36, TOP | 6 ± 0.8 years | The following factor correlates with EQ-5D score:   - Age   The following factors correlate with MCS (SF-36):   - Severity of craniocerebral injury - BDI values - Loss of job   The following factors correlate with PCS (SF-36):   - Age - Lower extremity injuries   The following factors correlate with the mental function-domain:   - Age - Severity of craniocerebral injury   The pain-domain is significantly associated with age.  Patients losing their job because of the trauma present with lower SF-36 and TOP values in all domains.  Patients reporting experience of financial losses due to the trauma report lower SF-36 and TOP values in all domains except for daily activities. |
| Falkenberg [43], 2017, retrospective cohort study, Germany | Patients (n = 324) with mental impairment (PTSD/MDD/both) were compared to patients without symptoms of mental impairment, ISS 21.5 ± 9.6, age 28.0 ± 11.7, 74.4% | Patients without symptoms of mental impairment (PTSD/MDD/both) | SF-12, HASPOC | 17.4 ± 4.9 years | Factors associated with mental impairment:   - GOS < 5 - Need for physical rehabilitation   Gender, age, or ISS do not affect psychological outcomes.  Patients with symptoms of impaired mental health report worse QoL more often (i.e., more loss of friends, financial loss, loss of partner, psychiatric treatment, and inability to work). Moreover, those patients tend to be less satisfied with their rehabilitation. |
| Macke [71], 2017, prospective cohort study, Germany | Patients with upper extremity trauma (n = 307), median ISS 22 (IQR 16)/mean ISS 21.2 (SD 9.6), mean age 27.4 ± 12.6, 72.6% | Outcomes for patients with and without a fracture of the upper extremity were compared | SF-12, HASPOC | 17.5 years (10-28) | There was no difference in the long-term outcome between those with upper extremity trauma and those without according to both instruments.  However, patients with a brachial plexus lesion had lower HRQoL, and less satisfaction with rehabilitation, longer duration of unemployment and more need for retraining. |
| Renovell-Ferrer, [76], 2017, retrospective cohort study, Spain | Patients (n = 86) with isolated calcaneus fractures were compared to polytraumatized patients sustaining calcaneus fractures – both had operated displaced intra-articular calcaneal fractures through open reduction and internal fixation (ORIF), results of patients with an ISS > 15 were investigated only (not further specified), mean age 48.4 (16-75), 71% | Polytrauma patients were compared to non-polytrauma patients | SF-36 | 48 months (15-62) | Psychiatric comorbidities predict worse HRQoL.  SF-36 scores were significantly lower in patients with more severe calcaneus trauma. |
| Weber [70], 2017, prospective cohort study, the USA | Patients with a Tegner Activity Scale (TAS), which characterizes the level of activity, below and above 5 were compared to each other to compare desired outcomes athletic and non-athletic patients (n = 465), mean ISS 21.6 ± 9.8, age 26 ± 11.5, 74.6% |  | SF-12, HASPOC | Mean follow-up duration of 17 ± 5 years | Injuries of the lower extremity were identified as the predictor that impact physical health the most. Patients identified injuries around the knee joint as primary cause of their posttraumatic impairment in sports. |
| Born [69], 2018, prospective cohort study, Switzerland | Patients having sustained traumatic brain injury (TBI) with ISS>15 (n = 199), mean ISS 22.01 ± 6.28, mean age 54.90 ± 18.49 years, 71.5% | Patients who had sustained major TBI (abbreviated injury scale, AIS head > 2) were compared with patients who had no or only mild TBI (AIS head ≤ 2). | QOLIBRI, EQ-5D, SF-36, TOP | 1 year follow-up | The following factors predict lower QOLIBRI, SF-36, EQ-5D and TOP-scores:   - Lower GCS (patients with a GCS <8 achieved the worst outcome in terms of QOLIBRI scores) |
| Fleischhacker [52] 2018, prospective cohort study, Germany | Severely injured patients (n = 168), mean ISS 23.6, age 42.6 ± 19.6 years, 77.4% |  | EQ-5D | 500 days follow-up interval (500-2500 days post-trauma) | There is an independent, U-shaped association between the frequency of extreme values of HRQOL and the time elapsed after injury.  The following factors are independent determinants for poor QoL (EQ-5D <=0.6):   - Intubation upon arrival in emergency room - Number of failing organs during ICU stay - Number of days elapsed after severe trauma injury   The following factors are independent determinants for poor QoL (EQ-VAS <=50):   - Predicted mortality according to RISC - Female gender - Number of days elapsed after injury |
| Leijdesdorff [55], 2018, retrospective cohort study, the Netherlands | Polytraumatized patients (n = 122; all with an ISS > 15), mean ISS 21.6 (SD 6.2), mean age 57.9 (SD 19.2), 66.4% | Dutch norm score | SF-36 | 15 (range 10-23) months after polytrauma | Worse physical health (PCS) is predicted by pre-existing comorbidity.  Worse mental health (MCS) is predicted by severe head injury.  Patients with reduced physical and/or mental health experience more general, physical, and mental fatigue and more reduced activity and motivation than patients without reduced HRQoL. Also, polytrauma patients with reduced QoL participate less often in social activities and experience more restrictions in daily life activities and are less satisfied with their social participation |
| Gross [148], 2018, prospective cohort study, Switzerland | Patients with traumatic brain injury (TBI) after major trauma (n = 326), mean ISS 15.50 (SD 7.24), mean age 53.47 (SD 19.22), 67.2% | Patients <65 years were compared to patients ≥ 65 years | EQ-5D, SF-36, TOP, QOLIBRI | 1-year post-trauma | - 1-year outcomes for younger and older adults following major trauma with TBI are comparable up to a patient age of 80 years. - The cognitive domain of the QOLIBRI demonstrated a trend towards a lower outcome in the elderly. - In the SF-36, two physical oriented subscores showed significantly lower values in the group of elderly. |
| Gross [149], 2018, prospective cohort study, Switzerland | Major trauma patients (n = 718), mean ISS 13.80 (SD 7.49), mean age 53.4 (SD 19.4), 66.9% |  | SF-36, EQ-5D, TOP | At least 1-year post-injury | - Age has a small impact on the longer-term outcomes of major trauma patients and is only relevant in least octogenarians - Largest associations of patient or trauma characteristics with longer-term outcome scores were the Injury Severity Score of the extremities with the PCS (R2=0.08) or the working capacity of employed patients (n=383; R2=0.04) - Patients aged 80 years or more presented with a significantly worse outcome compared to younger people in all overall and physical component scores. |
| Spijker [147], 2018, retrospective study, New Zealand | Adult survivors of major trauma (n = 65), mean ISS 25.4 (SD 10.1), mean age 46.3 (SD 21.2), 64.6% |  | EQ-5D-3L | 1-year post-injury | Poor outcomes were associated with:   - Younger age (16–50 years) - A history of psychiatric illness or substance use - ICU admission and spending >10 days in hospital |
| Spreadborough [146], 2018, cross-sectional cohort, UK | Major trauma patients who worked full-time pre-injury (n = 99), 55% of patients ISS >15, mean age 46.7 (SD 15.4), 74% |  | TOP | mean 23.8 months (SD 9.4) | Overall, patients with incomplete return to work had average scores below the cut-off points of the Trauma Outcome Profile, indicating increased levels of depression, anxiety, PTSD, pain and physical disabilities and reduced cognition, mental functioning, daily activities, and social interaction. In comparison, patients with complete return to work scored on average above the cut-off points for all these measures except PTSD. |
| van der Vliet [145], 2018, retrospective study, the Netherlands | Multiple trauma patients with Lisfranc and Chopart injuries who underwent ORIF (n = 40), median ISS 9 (4-17), mean age 33 (IQR 26-47), 68% | Dutch healthy reference population and Dutch reference trauma patients | EQ-VAS, EQ-5D-5L | not mentioned | For the EQ-5D, there was a significant relationship between concomitant injuries and lower long-term HRQoL.  No differences in EQ-5D scores between patients with and without concomitant calcaneal fractures were observed (p = 0.77). |
| Wad [59], 2018, prospective cohort study, Denmark | Severely injured patients (n = 63), median ISS 16 (range 4-33), age 35 years, 71.4% | Danish norm score | EQ-5D | 15 years after severe trauma | ISS is associated with HRQoL and ISS ≥ 16 predicts poorer HRQoL. |
| Angerpointner [57], 2019, prospective cohort study, Germany | Multiply injured patients (n = 501), mean ISS 21.9 ± 11.2, mean age 46.2 ± 19.1, 71% | Patients with an ISS <16 were compared to patients with an ISS>=16; data from a trauma centre level 1 were compared to data from a trauma centre level 2 | EQ-5D | After 6, 12 and 24 months | Patients with an ISS ≥ 16 have significantly lower EQ-5D-scores than patients with an ISS <16 at all 3 measurement points.  No difference in data was found between patients treated in a trauma centre level 1 and trauma centre level 2. |
| Beks [53], 2019, retrospective cohort study, the Netherlands | Patients with rib fixation for flail chest and/or multiple fractures of ribs (n = 166), median ISS 24 (IQR 18-34) for flail chest patients (n=66) and 21 (IQR 16-29) for multiple rib fracture patients (n = 99), median age 56 (IQR 47-69), 60.2% | Dutch norm score | EQ-5D | Median 3.1 years (IQR 2.4-5.1; range 1-7.5 years) in patients with flail chest, 4.4 years (IQR 3.4-5.9; range 1-7.6) for multiple rib fractures-patients | In patients with flail chest, the following factors associated significantly with lower EQ-5D score:   - Male sex - Sternum fracture   Patients with flail chest or multiple rib fractures reporting implant-related irritation at time of interview have significant lower QoL compared to patients without irritation. |
| Bott [38], 2019, prospective and retrospective cohort study, the UK | Cohort of patients with operatively treated unstable pelvic fractures (n = 75), mean ISS 17 (SD 10), mean age 15 (range 11-22), 70% | Age- and sex-matched scores for a UK population | EQ-5D, SF-36 | Mean time between surgery and follow-up: 15 years (11-22 years) | EQ-5D/SF-36: no statistically significant associations between age, ISS, presence of associated acetabular genitourinary injury or neurological injury.  SF-36: no significant association between fracture classification and outcome score for either PCS/MCS (for either OTA/AO or Young and Burgess classification system) |
| Gross [153], 2019, prospective longitudinal survey, Switzerland | Survivors of major trauma (n = 335), mean ISS 13.5 (SD 7.2), mean age 52.8 (SD 20.6), 70.7% |  | SF-36, EuroQoL, TOP, QOLIBRI | 1- and 2-years post-trauma | - A comparable increase over time was seen in both men and women for working capacity. - However, women, but not men, demonstrated improvements in the second year after injury. In addition, female patients achieved a higher level of outcome 2 y after trauma than men did on most of the evaluation instruments. |
| Llaquet [152], 2019, prospective follow-up observational study, Spain | Major trauma patients admitted to the ICU (n = 200), mean ISS 15.2 (SD 7.9), mean age 47.8 (SD 19.9), 72.5% | Normative Spanish population values | EQ-5D-5L | 3, 6 and 12 months after injury | Determinants for worse EQ-US scores at 12 months were:   - Female gender - Age ≥ 55 years - Injury severity score (ISS) ≥ 25 - Severe extremity injury - Unskilled employment |
| Rainer [150], 2019, prospective cohort study, China | Moderate to major trauma patients (n = 119), 58.8% of the population ISS ≥ 16, mean age 47.7 (SD 17.7), 75.6% | Hong Kong population norms | SF-36 | 5 years post-trauma | After injury, further recovery was still evident for MCS in patients aged under 65 years for up to five years. |
| Saengniam [81], 2019, cross-sectional, Thailand | Recently (<5 years) multiply injured patients admitted to tertiary hospitals (n = 106), mean ISS 19.15 ± 11.20, mean age 37.53 ± 13.54, 84% | NA | TOP |  | The following factors predict overall poor HRQoL:   - High pain intensity (most influential factor) - Disability - Poor emotional coping   The following factor predicts a positive overall HRQoL:   - Resilience (defined as the ability to adapt easily to adversity). |
| Van der Vliet [130], 2019, retrospective cohort study, the Netherlands | Patients with distal radius fracture(s) (n = 65), median ISS 22 (IQR 19-29), median age 45 (IQR 26-60), 69% | Compared to patients who sustained low-energy distal radius fractures as their only injury (monotrauma patients) | EQ-5D | Median 5 years (IQR 4-7 years) | - Polytrauma (defined as ISS ≥ 16) is significantly associated with lower HRQOL and modest poorer wrist function. - Worse HRQOL scores correlated with worse wrist function according to the QuickDASH score - Patients who sustained distal radius fractures in the context of polytrauma or high-energy trauma reported slightly worse EQ-5D-3L scores |
| Velmahos [151], 2019, retrospective cohort study, the USA | Major trauma patients (n = 1238), all with ISS ≥ 9, 55.3% aged below 65 years, 55.2% |  | Trauma Quality of Life questionnaire | 1 year after injury | Independent predictors for presence of daily pain 1-year post-injury:   - Drug use disorder (OR 1.73, 95% CI 1.03-2.9) - Alcohol use disorder (OR 1.64, 95% CI 1.1-2.45) - Hospital days ≥ 5 days (OR 1.62, 95% CI 1.27-2.08) - Age < 65 years (OR 1.58, 95% CI 1.22-2.06) - Orthopaedic operation (OR 1.55, 95% CI 1.15-2.1) - Low education (OR 1.53, 95% CI 1.18-1.96) - Extremity injury (OR 1.52, 95% CI 1.11-2.09)   Independent predictors for use of pain medication 1-year post-injury:   - Alcohol use disorder (OR 1.97, 95% CI 1.17-3.32) - Drug use disorder (OR 1.91, 95% CI 1.1-3.34) - Fall (OR 1.89, 95% CI 1.32-2.68) - Unemployed (OR 1.73, 95% CI 1.27-2.37) - Smoker (OR 1.65, 95% CI 1.07-2.53) - Hospital stay ≥ 5 days (OR 1.6, 95% CI 1.2-2.14) - Chest injury (OR 1.53, 95% CI 1.06-2.21) - Low education (OR 1.39, 95% CI 1.03-1.87) - 1 or more comorbidities (OR 0.72, 95% CI 0.54-0.96) |
| Freigang [156], 2020, prospective cohort study, Germany | Severely traumatized patients (n = 405), mean ISS 25.6 (SD 10), median age of group <65 years 43.0 (24.3-53.0), median age of group ≥ 65 years 73.0 (IQR 70.0-78.0), 72% | Patients < 65 years were compared to patients ≥ 65 years; overall scores were compared to general German population norms | EQ-5D-3L | 6-, 12- and 24-months post-trauma | Worse HRQoL outcomes were predicted by:   - Age ≥ 65 years (compared to patients aged <65 years) - Increasing AIS extremities |
| Martino [85], 2020, retrospective cross-sectional study, Italy | Major trauma patients admitted to level I trauma centre ICU (n = 428), median ISS 27 (IQR 15), mean age 39.1 (SD 20.1)/median age 26.5 (IQR 33), 74.3% | NA | EQ-5D, **PCL** | 1-year post-injury | - HRQoL is strongly correlated with global level of functioning/   functional independence |
| Simmel [154], 2020, prospective cohort study, Germany | Polytraumatized patients (n = 84), all with an ISS ≥ 25, mean age 37.3 (SD 11.5; range 18-59), 78.6% |  | POLO-chart | mean 67.7 (SD 20.0; range 38-108) months post-injury | Predictors for return to work were:   - Age - Duration of treatment in ICU - Time between admission and follow-up - Self-reported general health   Pre-existing comorbidities or other stressful events do not contribute to the prediction of RTW. |
| Tipping [155], 2020, prospective observational study, Australia | Major trauma patients above 50 years and admitted to the ICU (n = 138), mean ISS 20.99 (SD 10.17), mean age 67.61 (SD 10.41), 79.7% | Frail patients were compared to non-frail patients | EQ-5D-5L | 6- and 12-months post-injury | Frailty is a predictor of poor outcomes in critically ill trauma patients. |
| Castillo-Angeles [159], 2021, prospective cohort study, the USA | Moderate to severely injured patients due to violence (n = 176), all ISS ≥ 9, mean age 34.4 (SD 12.5), 85% | Non-violent trauma survivors | Trauma QoL questionnaire | 6-12 months post-violent event | A violent trauma mechanism is significantly associated with:   - PTSD   A violent trauma mechanism is not significantly associated with:   - Chronic pain - Return to work - Functional outcomes |
| Giummarra [160], 2021, retrospective cohort study, Australia | Patients with penetrating injury following major trauma (n = 208), all with an ISS >12, average age 38.9 (SD 15.7), | General Australian population values | EQ-5D-3L | 6, 12, 24 months post-injury | Worse HRQoL was associated with:   - Middle-older age - Female sex - Unemployment - Pre-injury disability - Comorbid conditions - Assault - Firearm injury compared to cutting/piercing injuries |
| Curtis [161], 2022, retrospective cohort study, Australia | Children (< 16 years) with major trauma (n = 510), median ISS 9 (IQR 9-16), mean age 6.7 (SD 5.1), 71.8% |  | PedsQoL, EQ-5D-Y | 12 months post-injury | Worse psychosocial health at 12 months was associated with:   - Hospital length of stay - Number of body regions injured - Polytrauma   Worse physical outcome was associated with:   - Hospital length of stay - ICU admission   Polytraumatized patients presented with worse QoL than non-polytraumatized patients. |
| van Ditshuizen [157], 2022, retrospective cohort study, the Netherlands | Major trauma patients (n = 182), median ISS 20 (IQR 17-25), median age 52 (29-67), 64% | Dutch population values | EQ-5D-5L | 1-year post-trauma | Predictors for worse HRQoL were:   - Age groups 18–55 yrs (compared to 0-17 yrs) - Having comorbidities - ISS ≥ 25 compared to ISS 16–24 - Severe lower extremity injury (MAIS ≥ 3) compared with moderate (MAIS 1–2) lower extremity injuries or patients without lower extremity injuries - Severe spine injuries (MAIS ≥ 3) compared to patients without spine injuries - Severe head injuries (MAIS ≥ 3) compared with moderate (MAIS 1–2) or no head injuries - Moderate (MAIS 1–2) and severe face injuries (MAIS ≥ 3)   Patients with an ISS ≥ 25 were less likely to return to work than patients with an ISS 16-24. |
| Zeelenberg [158], 2022, retrospective cohort study, the Netherlands | Polytrauma patients (n = 598), median ISS 22 (IQR 18-29), median age 51 (IQR 37-62), 70.6% | Patients with upper extremity injury (UEI) were compared to patients without UEI | SF-36, EuroQoL-5D | median 75 (IQR 50-102) months | - Patients with upper-extremity injuries showed a minor increase in disability in the Disabilities of the Arm, Shoulder and Hand score than patients without upper-extremity injuries. |
